# Chemical pattern visualization in 2D – the SMARTSviewer

**DOI:** 10.1186/1758-2946-3-S1-O12

**Published:** 2011-04-19

**Authors:** Karen Schomburg, Hans-Christian Ehrlich, Katrin Stierand, Matthias Rarey

**Affiliations:** 1 ZBH – Centre for Bioinformatics, University of Hamburg, Bundesstr. 43, 20146 Hamburg, Germany

## 

Chemical patterns are essential for various fields of chemical, chemoinformatic and pharmaceutical applications. So far, representations of chemical patterns are limited to linear molecular pattern languages like SMARTS[1]. As these languages are designed for computational efficiency, they are often hardly human readable. In order to improve the usability of chemical patterns for scientists without expert knowledge of one of these languages, we present a visual representation of chemical patterns similar to structure diagrams.

While molecules can also be represented by systematic names, the means of communication of compounds among scientists is the visual representation of 2D structure diagrams. Therefore, we propose a depiction of chemical patterns based on the common standard concept of structure diagrams. As chemical patterns denote descriptions of chemical features, the concept of structure diagrams is extended with graphical elements to depict property descriptions and logic combinations of chemical features. The aim of the depiction is to provide an overview of the specified features as well as to highlight similarities and differences among patterns.

As a first application of the new visualization concept we developed the SMARTSviewer. The tool converts a pattern in form of a SMARTS string into a graphic representation. Along with the graphic depiction, the tool produces a legend explaining the graphic symbols and meaning of the features described in the pattern. The SMARTSviewer is openly accessible [2,3].

Since commonly accepted visual depictions have to evolve from the needs of the users, we hope to initiate a discussion based on the concept we introduce.
